# Marginal Misfit of 3D-Printed (Selective Laser Sintered), CAD-CAM and Lost Wax Technique Cobalt Chromium Copings with Shoulder and Chamfer Finish Lines: An In-Vitro Study

**DOI:** 10.3390/medicina58101313

**Published:** 2022-09-20

**Authors:** Samar Al-Saleh, Fahim Vohra, Shabab M. Albogami, Nawaf M. Alkhammash, Mohammed A. Alnashwan, Naif S. Almutairi, Khalid A. Aali, Mohammed Alrabiah, Tariq Abduljabbar

**Affiliations:** 1 Department of Prosthetic Dental Science, College of Dentistry, King Saud University, Riyadh 11545, Saudi Arabia; 2 Department of General Dentistry, College of Dentistry, King Saud University, Riyadh 11545, Saudi Arabia

**Keywords:** misfit, marginal opening, lost wax technique, selective laser melting, CAD-CAM

## Abstract

*Background and Objectives:* The aim was to compare the Misfit of 3D-Printed, Selective laser melting (SLM), milled (Computer aided design-Computer aided manufacture CAD-CAM) and Lost wax technique (LWT) fabricated Cobalt chromium (CoCr) alloy copings on shoulder (SH), radial shoulder (R-SH) and chamfer (CH) finish line configuration. *Materials and Methods:* Ninety resin, second maxillary premolar teeth were prepared for metal-ceramic crowns, equally divided into (*n* = 30) SH, R-SH and CH margin preparations. For each preparation design (SH, R-SH and CH), CoCr copings were prepared using SLM, CAD-CAM and LWT. This resulted in nine study groups with 10 CoCr copings each. The marginal misfit of specimens was assessed with a high-resolution digital microscope. Misfit was evaluated in vertical and horizontal dimensions in μm. Data were analyzed using ANOVA and a post hoc multiple comparisons test. *Results:* For vertical misfit, the highest was observed in SLM samples with chamfer margin (167.96 ± 24.1), and the least was shown by CAD-CAM samples with radial shoulder (58.8 ± 12.53). CAD-CAM and shoulder margins showed the least vertical misfit. For horizontal misfit, the maximum was observed in SLM samples with shoulder margin (137.94 ± 37.85) and the least by LWT samples with chamfer (89.38 ± 14.81). Chamfer margins and LWT samples showed the least horizontal misfit among the group samples. Fabrication technique and finish line design play a critical role in reducing the marginal misfit of CoCr copings. *Conclusions:* For vertical misfit, SLM copings showed poor outcomes compared to CAD-CAM specimens, however comparable outcomes to Cast specimens. SLM copings showed comparable horizontal misfit outcomes to CAD-CAM specimens and low misfit compared to Cast copings, respectively. Vertical misfit was low with shoulder margins, and horizontal misfit was better with chamfer marginal configuration.

## 1. Introduction

Indirect restorations supported by teeth and implants are commonly employed for rehabilitation of lost or congenitally missing teeth. Metal-ceramic prosthesis is still the main stay of indirect crowns and fixed partial denture restorations, providing reliable long-term function, comfort and esthetic replacement [1]. Factors related to failure of metal-ceramic crowns include poor marginal fit, oral hygiene, endodontic or pulpal failure and mechanical fracture [2,3]. Marginal misfit and opening result in plaque accumulation, discoloration, gingiva irritation, cement dissolution, recurrent caries and periodontal disease [4]. Misfit is defined as the vertical and horizontal gap between the prepared tooth surface and the surface of metal coping. Therefore, achieving a robust seal and minimum marginal gap at the restorative margin is the operators’ goal in dental prosthetic treatment.

Factors affecting the marginal misfit of metal substructure of metal-ceramic crowns include fabrication techniques (Computer aided design- Computer aided manufacture- CAD-CAM), lost wax technique (LWT), copy-milling), ceramic firing, restoration type, margin design, impression materials, die materials and processing techniques [5]. The most common fabrication techniques are conventional casting and subtractive manufacturing technologies (SMT) including CAD-CAM [6,7]. However, these technologies present a number of manufacturing limitations. Conventional casting is a sensitive technique with multiple steps, being time-consuming, operator and material dependent; and therefore is prone to errors [8,9,10]. CAD-CAM technology offers increased accuracy and efficiency, reduced time, labor and cost of production and reproducibility; however, wastage of material and finite resolution limits its ability to fulfill misfit goals [8,11]. In addition, evolution in CAD-CAM technology has resulted in a myriad of milling systems, which show misfit outcomes ranging from 16 μm to 100 μm [6]. In recent times, introduction of additive manufacturing techniques (AMT) has offered a reduction of errors and inaccuracies with laboratory procedures during fabrication of prosthesis. The Selective laser melting (SLM) technology is one of the popular AMTs, which is based on a build platform with metal powder particles being melted using a heat source such as a laser or electron beam to form 3D objects, layer by layer [12]. SLM is introduced in prosthodontics, including fabrication of crowns, metal frameworks, crown copings, post and core, removable partial dentures and implant-abutments [12,13,14,15,16,17,18,19]. In a study by Castillo-Oyague et al., SLM CoCr copings showed best vertical adaptation in comparison with cast CoCr copings [17]. However, SLM CoCr fixed partial denture (FPD) frameworks have shown higher marginal and internal gaps than cast NiCr FPD frameworks [20].

A critical factor influencing marginal misfit is tooth preparation finish line design [21,22,23]. It is suggested that shoulder margins with a bevel of metal-ceramic castings showed better marginal fit compared to a 90° shoulder [23]. However, Shillingburg has suggested that shoulder margins show less marginal opening compared to chamfer margins after ceramic firing in metal-ceramic crowns [21]. The influence of finish line configuration on marginal misfit is due to the limitations of the fabrication technique and technical errors. Therefore, it is believed that the influence of finish line configuration of tooth preparations for metal-ceramic in the presence of improved fabrication methods will be reduced. In addition, studies assessing the influence of finish line configurations on metal copings fabricated with the SLM technique are limited. It is hypothesized that SLM fabricated metal copings will show no influence of finish line configuration (shoulder (SH), radial shoulder (R-SH) and chamfer (CH)), on their marginal misfit. Furthermore, these SLM samples will show comparable marginal misfit to CAD-CAM and cast copings. Therefore, the aim of the present study was to compare the effect of shoulder, radial shoulder and chamfer finish line configuration of metal copings fabricated using SLM, CAD-CAM and LWT on their marginal misfit.

## 2. Materials and Methods

### 2.1. Ethical Considerations

The study protocol was evaluated by the college of dentistry research center and was formally approved (IR-0394-20 August 2022). The study did not include clinical patients or human tissue in any part of the experiments. The study protocol was reported in accordance with the checklist of reporting in-vitro studies (CRIS guidelines). At a level of significance alpha 0.05, with effect size 0.46 and power 0.85, for two independent variables, the total sample size was evaluated to be 90 overall, with 10 samples in each group (One way ANOVA). A similar sample size has been employed in a similar previous study [24].

### 2.2. Tooth Preparation

**Figure 1:**
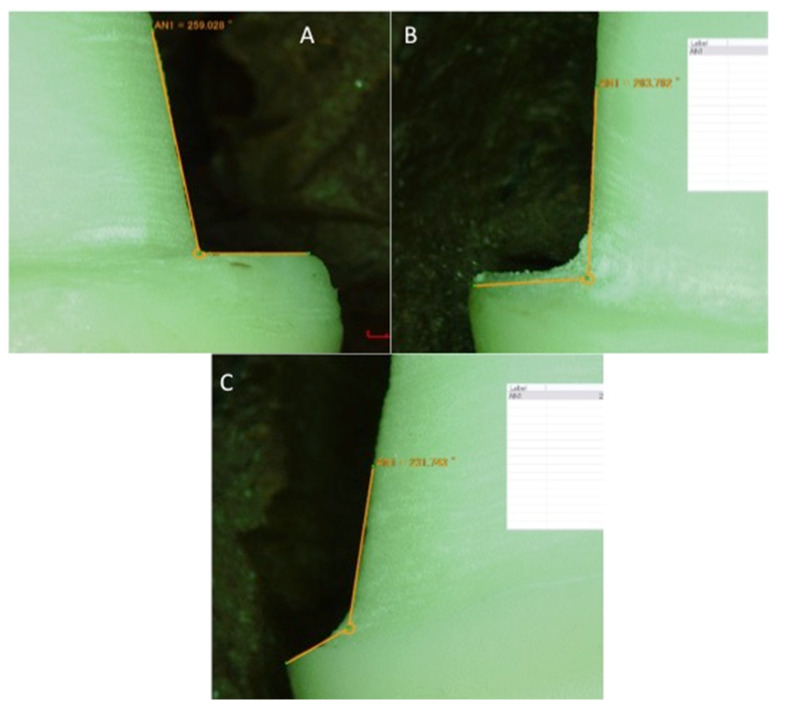
Prepared marginal configurations. (**A**) shoulder, (Yellow line indication 90° angle) (**B**) radial shoulder (yellow line indicating 90° with rounded line angle, (**C**) chamfer (Yellow line indication 135° angle).

**Figure 2:**
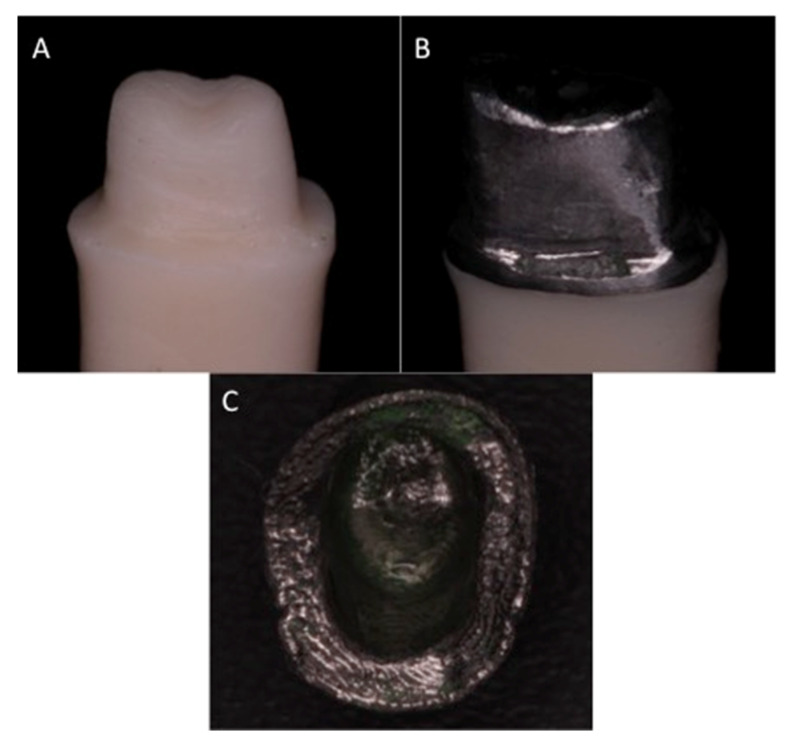
(**A**) tooth preparation, (**B**) lost wax technique coping, (**C**) Selective laser melting (SLM) coping.

In this in-vitro experiment, ten samples were included in each group based on the recommendations of previous study (a). Ninety resin maxillary second premolar teeth (Typodont teeth, FSFF, NY, USA) were obtained and mounted in resin acrylic (Clear Orthodontic resin, Dentsply Sirona, Charlotte, NC, USA) using a dental surveyor (Dentsply Ney Surveyor, Dentsply Sirona, Charlotte, NC, USA). A putty index (Aquasil Putty, Dentsply Sirona, Charlotte, NC, USA) was made for each tooth and all mounted teeth received guided preparations for a metal-ceramic crown by two experienced prosthodontists. The preparation included reductions of 1.5 mm labial with two planes, 1.5 non-functional and 2 mm functional cusp and 1 mm lingual and variable finish lines (shoulder, radial shoulder and chamfer). The tooth preparations were made using a high-speed airotor handpiece (Handpiece-LED, Kavo, Manchester, UK) operating at 180,000 rpm with copious water irrigation and diamond burs (Mani, Tokyo, Japan). The finish lines in the study groups were prepared with modified chamfer diamond bur (chamfer-FG-878-016, Diamond, Henry Schein, Melville, NY, USA), shoulder bur (FG-847 KR-016, Diamond, Henry Schein, Melville, NY, USA) and rounded shoulder (FG-845 KR-016 Diamond, Henry Schein, Melville, NY, USA) burs. The prepared teeth were divided into three major groups (*n* = 30) based on the type of finish lines (Shoulder, Chamfer and radial shoulder) with 30 samples each. The final finish line preparations of the samples were verified using a digital microscope (Figure 1 and Figure 2A).

### 2.3. Study Groups (Table 1)

Based on the margin design and coping fabrication techniques, the study had the following groups:

Shoulder (SH) (*n* = 30);

SH-SLM: Ten CoCr metal copings were prepared using the selective laser melting (SLM) technique;

SH-CAD-CAM: Ten CoCr metal copings were prepared using computer aided design and computer aided manufacturing (CAD-CAM) techniques;

SH-CAST: Ten CoCr metal copings were prepared using a lost wax technique (LWT) technique;

Radial-Shoulder (R-SH) (*n* = 30);

R-SH-SLM: Ten CoCr metal copings were prepared using selective laser melting (SLM) technique;

R-SH- CAD-CAM: Ten CoCr metal copings were prepared using computer aided design and computer aided manufacturing (CAD-CAM) techniques;

R-SH-CAST: Ten CoCr metal copings were prepared using lost wax technique (LWT);

Chamfer (CH) (*n* = 30);

CH-SLM: Ten CoCr metal copings were prepared using a selective laser melting (SLM) technique;

CH-CAD-CAM: Ten CoCr metal copings were prepared using computer aided design and computer aided manufacturing (CAD-CAM) techniques;

CH-CAST: Ten CoCr metal copings were prepared using a lost wax technique (LWT).

**Table 1:** Study groups.

Margin Type	Fabrication Technique	Study Groups
Shoulder (SH)	SLM	SH-SLM
	CAD-CAM	SH-CAD-CAM
	CAST	SH-CAST
Radial-Shoulder (R-SH)	SLM	R-SH-SLM
	CAD-CAM	R-SH-CAD-CAM
	CAST	R-SH-CAST
Chamfer (CH)	SLM	CH-SLM
	CAD-CAM	CH-CAD-CAM
	CAST	CH-CAST

SLM: Selective laser melting; CAD-CAM: Computer aided design-computer aided manufacture; CAST: Lost wax technique/Casting.

### 2.4. Coping Preparation

Thirty copings in each margin group (SH, R-SH and CH) on their respective tooth preparations were prepared using CAD-CAM, SLM and LWT.

### 2.5. CAD-CAM

The prepared teeth were scanned using a Cercon Eye scanner (DeguDent GmbH, Hanau, Germany). The copings (*n* = 30) were designed using Cercon Art according to the following dimensions, cement gap of 0.05 mm starting 1 mm above the margin; and margin reinforcement of 0.01 mm. The axial walls were 1 mm thick, with 0.5 mm occlusal thickness. The coping design was saved in an STL file. Cercon Brain (DeguDent GmbH, Hanau, Germany) milling machine was used for coping fabrication, using Ceramill Sintron alloy blanks (CoCr-Amann Girrbach AG Herrschaftswiese, Koblach, Austria) as per manufacturer recommendations.

The design of each coping was selected from the display, and on complete milling, blanks were removed. The milled copings were further sintered in Ceramill Argotherm II (Processing furnace) (CoCr-Amann Girrbach AGHerrschaftswiesen) and sintered for 170 min at 1600 °C.

### 2.6. Selective Laser Melting (SLM)

The dimensions of the copings in all groups were standardized. To fabricate SLM copings, the design STL file was transferred to a Concept Laser Machine (mlab metal laser melting system; GE Additive company, Boston, MA, USA) with standard parameters. A CoCr alloy (Starbond Easy Powder 30; GmbH, Mainz, Germany), containing Co 61%, Cr 27.5%, W 8.5%, Si 1.6%, C, Fe and Mn < 1% and an alloy powder grain size of +10/−30 µm was utilized to fabricate the copings (Figure 2C).

The three-dimensional coping model was vertically positioned, and support was designed and attached to coronal part of the copings in the software. The printing was performed in nitrogen and argon atmosphere and a 100 W ytterbium (Yb) laser beam hit melted the powder layer resulting in powder fusion. Each layer of fusion was 20 μm, and the building platform moved down with deposition of a new powder layer during the process. This process repeated itself until the coping was fabricated and sintered.

### 2.7. Lost Wax Technique (LWT)

A double layer of die spacer (Platzhalterlack; Gramm GmbH & Co. KG, Muhnhausen, Germany) was applied 1 mm above the preparation margin. A wax up (Blue inlay casting wax, Kerr Corporation, Brea, CA, USA) was performed by an experienced senior technician. Each wax pattern was replicated to have similar dimensions to the milled metal alloy copings (Section 2.5). The wax patterns were sprued and invested using phosphate-bonded investment (Fast Fire 15 investment; Whip Mix, Louisville, KY, USA). Burnout of the wax patterns was performed using a heat furnace (PROGRAMIX 50, Ugin’Dentaire, Seyssinet-Pariset, France) at 900 °C. Casting was performed (FORNAX 35E^®^, BEGO, Bremen, Germany) at 1500 °C with CoCr alloy (Wirebond^®^C; BEGO, Bremen, Germany) (composition Co 63.3% Cr 24.8% W 5.3% Mo 5.1% Si 1.0%) followed by divesting and ultrasonic cleaning (Figure 2B).

### 2.8. Assessment of Marginal Misfit

The marginal misfit of the CoCr copings fabricated using CAD-CAM, SLM and LWT methods was evaluated using a high magnification digital microscope (Hirox KH-7700). The CoCr copings were secured on the mounted teeth using metal screw clamp apparatus for performing reliable measurements with stable specimens. The digital microscope was calibrated at the beginning of the measuring session and specimens were positioned on the stage using a positioning jig for reproducible positioning of the samples. The vertical marginal misfit was measured as a gap between the lowest point of the copings and highest outer margin on the preparation. The horizontal marginal misfit was measured as a measurement from the outer most point on the coping margin to the outer most point on the preparation margin. The measurements for misfit were performed by selecting a point on the margin and dragging the cursor to the second point, resulting in a misfit number in microns (μm). The measurements were made on both buccal and lingual margins at six points followed by a calculation of mean value. The operator examining the misfit was calibrated using the ImageJ software (National institute of health, NIH, Java 1.8. 0_45 (64-bit), Bethesda, MD, USA).

### 2.9. Statistical Analysis

Statistical analysis was performed using statistical program for social sciences (SPSS, version 23, IBM, Armonk, NY, USA). The data obtained were assessed for normality testing using the Kolmogorov–Smirnov test. A *p*-value of less than 0.05 was considered statistically significant. Means and standard deviations were compared using Analysis of Variance (ANOVA) and Tukey–Kramer multiple comparisons test.

## 3. Results

**Figure 3:**
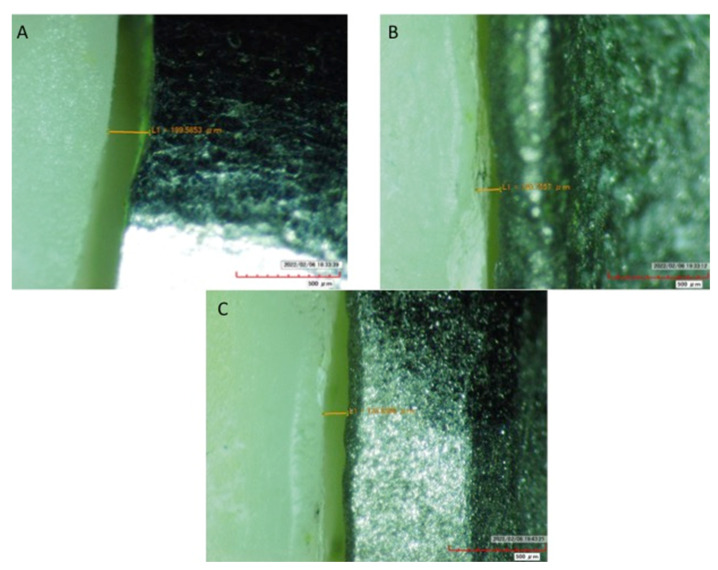
Vertical misfit assessment at standard magnification SLM (Yellow line indicating magnitude of misfit in each figure). (**A**) Radial Shoulder (mean: 166.96), (**B**) Chamfer (mean. 167.96), (**C**) Shoulder (mean. 105.20).

The misfit data among the study groups were normally distributed. For vertical misfit, the highest was observed in SLM samples with chamfer margin (167.96 ± 24.1), and the least was shown by CAD-CAM samples with radial shoulder (58.8 ± 12.53). Among SLM specimens, shoulder margin showed a significantly lower misfit (105.20 ± 26.22) (*p* < 0.05), compared to radial shoulder (166.96 ± 27.68) and chamfer margins (178.96 ± 24.1) (Table 2). For CAD-CAM specimens, shoulder (68.64 ± 14.10) and radial shoulder (58.80 ± 12.53) samples showed comparable misfit (*p* > 0.05), which was significantly smaller than chamfer (102.71 ± 18.01) margins (*p* < 0.05). Among the cast samples, shoulder margins showed lower misfit (124.81 ± 22.0) compared to radial shoulder (161.62 ± 29.44) and chamfer margins (163.30 ± 27.57) (*p* > 0.05). Among all three-margin types (SH, R-SH and CH), CAD-CAM samples showed significantly lower misfit compared to SLM and Cast samples (*p* < 0.05) (Table 2 and Figure 3). However, for all three-margin types, SLM and LWT samples showed comparable misfit values. Overall, CAD-CAM and shoulder margins showed the least misfit among the group samples.

**Figure 4:**
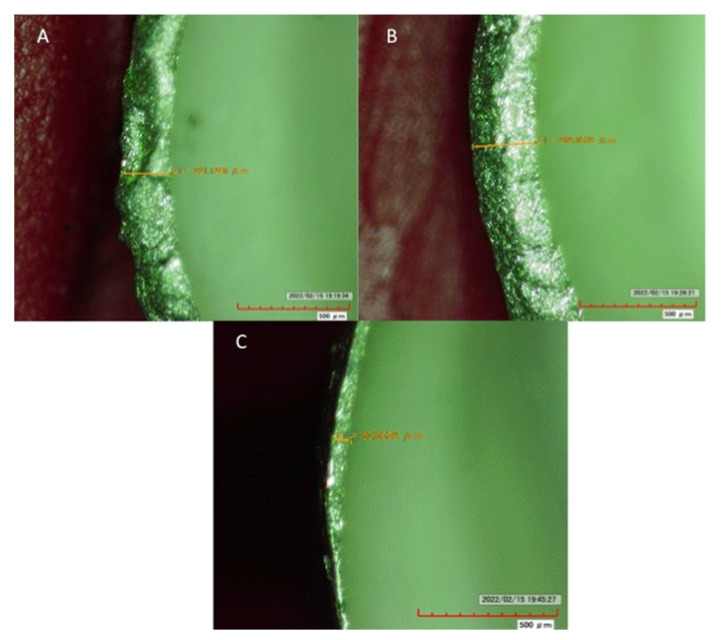
Horizontal misfit assessment at standard magnification for SLM samples (yellow line indicates extent of misfit in each figure). (**A**) Radial Shoulder (mean. 133.39), (**B**) Shoulder (mean. 137.94), (**C**) Chamfer (mean. 116.71).

For horizontal misfit, the highest misfit was observed in SLM samples with shoulder margin (137.94 ± 37.85), and the least was shown by LWT samples with chamfer (89.38 ± 14.81). Among SLM specimens, shoulder margin showed itself to be higher (137.94 ± 37.85) however having comparable misfit to radial shoulder (133.39 ± 20.87) and chamfer (116.71 ± 27.67) margins (Table 3 and Figure 4). For CAD-CAM specimens, shoulder margin (135.00 ± 13.56) showed high misfit, which was comparable (*p* > 0.05) to radial shoulder (114.91 ± 15.29), but significantly higher (*p* < 0.05) than chamfer margins (95.31 ± 18.31). Among the cast samples, although chamfer margin showed lower misfit (89.38 ± 14.81), all margin types showed comparable misfit statistically (*p* > 0.05). Among all three margin types (SH, R-SH and CH), cast samples showed significantly lower misfit compared to SLM (*p* < 0.05) (Table 3). For horizontal misfit, SLM showed comparable misfit to CAD-CAM samples (*p* > 0.05). Overall, chamfer margins and LWT samples showed the least horizontal misfit among the group samples. Type of fabrication technique had a greater influence on horizontal misfit than margin types. Both fabrication technique (*p* < 0.05) and the type of margin configuration (*p* < 0.05) had an overall significant influence on the vertical and horizontal misfit of coping specimens.

## 4. Discussion

The present study was based on the hypothesis that SLM fabricated metal copings will show no influence of finish line configuration (shoulder (SH), radial shoulder (R-SH) and chamfer (CH)), on their marginal misfit; and SLM samples will show comparable marginal misfit to CAD-CAM and LWT copings. It was observed that the finish line had a significant effect on the marginal misfit among SLM samples, and vertical and horizontal misfit among SLM copings were significantly compromised compared to CAD-CAM and LWT copings, respectively. Therefore, both hypotheses were rejected. The significant difference in marginal misfit of metal copings can be attributed to the limitations of the fabrication technique, geometric configuration of the finish lines and misfit evaluation technique.

In the present study, marginal misfit was evaluated using a highly sensitive digital microscope (Hirox-KH 7700). The digital microscope employed supports a magnification of more than 500× and allows for the easing of sample evaluation without sample sectioning, destruction or coating. The digital microscope allows image capturing of the samples at different orientations, with efficient and convenient procedures. Previous studies have used a digital microscope to evaluate the marginal misfit of indirect restorations [25]. Each sample was secured using a screw assisted locking device to reliably evaluate marginal misfit. In addition, inter-examiner reliability using kappa scores (0.90) was evaluated to standardize the technique for misfit. Interestingly, the samples were not cemented in the present study, to prevent the influence of fluid dynamics and cement filtration on marginal misfit. In addition, the presence of a cement layer requires maintaining a standard load during coping seating to avoid errors in in-vitro studies [6]. Interestingly, ceramic firing on metal copings may compromise the marginal fit of metal-ceramic crowns; however, it was not performed in the present study, to avoid introducing inaccuracies in evaluating the effect of technique and finish lines on misfit [5]. However, further studies investigating the influence of ceramic firing on the marginal misfit of SLM fabricated metal alloy copings are recommended.

An ideal marginal fit of indirect restorations does not exist, and continuous development of newer materials and methods for indirect restorations and cements has resulted in a lack of a gold standard for acceptable clinical and biological fit. Classic literature suggests that clinically detectable marginal misfit for sub-gingival and supra-gingival finish lines is 34–119 μm and 2–51 μm, respectively [26]. In addition, the American Dental Association (ADA) proposed a limit of cement film thickness to be 25 μm and 40 μm for type I and type II luting agents [27]. However, misfit values of less than 80 μm are difficult to detect clinically and, therefore, multiple authors have suggested marginal discrepancy of up to 150 μm to be clinically acceptable [28,29]. In the present study, the majority of misfit values were within the acceptable range (150 μm).

In the present study, SLM copings showed higher vertical misfit compared to CAD-CAM copings; however, they were comparable to LWT copings. A possible explanation for the relatively higher misfit of SLM copings is related to the surface topography and roughness of SLM samples. In a study by Li et al. [30], it was suggested that the SLM method results in rough restorative surface as it has a staircase effect due to the increase of the number of deposition layers, attachment profile of partially melted particles of powder on the external surface, large powder size, and the presence of porosity and un-melted regions related to parameters that control defect formation. It is the author’s opinion that the surface micro roughness and irregularities of SLM copings are correlated to the increased misfit findings in the present study. The LWT copings also showed increased misfit compared to CAD-CAM samples, due to the limitations of the casting technique, including wax expansion, alloy shrinkage, oxide layer and porosity, in comparison to SLM and CAD-CAM methods [31]. The outcomes of the present study show comparable vertical marginal misfit among LWT (124–163 μm) and SLM (105–167 μm) copings; these findings are similar to the earlier study by Huang et al. [15]. This suggests that SLM copings have the potential to be used as an alternative to LWT copings in restorative tooth replacement. Interestingly, the overall horizontal misfit, which represents overhang or ledge restorative margins, was lower compared to vertical misfit in the different fabrication groups (LWT, CAD-CAM and SLM) (horizontal misfit 93 to 137 μm, vertical misfit, 58–167 μm). LWT showed low horizontal misfit outcomes compared to SLM and CAD-CAM copings. The LWT process involved waxing up of copings, which undergo expansion; however, there is predominant alloy shrinkage in LWT, it can increase the vertical gap; however, it will reduce the horizontal outer periphery of the copings, resulting in reduced horizontal misfit of coping margin to the preparation finish line. Therefore, although the horizontal misfit measures for SLM and CAD-CAM copings were within the acceptable range (95–137 μm), LWT copings showed even lower misfit (89–100 μm).

Finish line types assessed included shoulder radial shoulder and chamfer in the present study. Overall, vertical misfit was low in shoulder margins in all fabrication method copings. However, chamfer margins showed low horizontal misfit in all copings (SLM, LWT and CAD-CAM). Previous studies have suggested that chamfer margins with bevel show better marginal sealing compared to shoulder margins; however, occlusal seating is relatively better for 90° shoulder margins [32]. However, a classic study by Gavelis et al. reported that a 90° shoulder finish line had lower misfit than a 45° and 30° shoulder [33,34]. Therefore, marginal discrepancies are greatly reduced where accurate seating is achieved, and horizontal margins including shoulder and chamfers appear superior in this respect.

The findings of the present study suggest that fabrication technique and finish line design play a critical role in reducing the marginal misfit of CoCr copings. SLM copings showed comparable misfit outcomes to CAD-CAM and LWT methods for all three finish line configurations. It is known that ceramic firing of metal copings compromises marginal fit [5] of metal-ceramic crowns. However, in the present study, porcelain application was not performed, and ceramics firing of SLM copings may show variable outcomes. Moreover, Ti and gold alloys are available for fabrication of copings and have different casting and sintering properties than CoCr, potentially improving the accuracy of casting. Therefore, the present study findings can only be considered for CoCr material with the SLM technique, and further studies comparing different alloy material copings fabricated with SLM with ceramic firing are recommended. It is the author’s opinion that randomized controlled trials comparing metal-ceramic crowns and fixed partial dentures, fabricated with the SLM technique to clinically validate present study in-vitro findings, are warranted.

## 5. Conclusions

For vertical misfit, SLM copings showed poor outcomes compared to CAD-CAM specimens, with comparable outcomes to Cast specimens. SLM copings showed comparable horizontal misfit outcomes to CAD-CAM specimens and low misfit compared to cast copings, respectively. Vertical misfit was low with shoulder margins, and horizontal misfit was better with chamfer marginal configuration. Fabrication technique and finish line design play a critical role in reducing the marginal misfit of CoCr coping.

## Figures and Tables

**Table 2:** Mean and standard deviations of vertical misfit (μm) among the study groups.

Margin Type	SLM	*p*-Value *	CAD-CAM	*p*-Value *	LWT	*p*-Value *
	Mean (SD) (μm)		Mean (SD) (μm)		Mean (SD) (μm)	
Shoulder (SH)	105.20 (26.22) ^a A^	<0.01	68.64 (14.10) ^a B^	<0.01	124.81 (22.0) ^a C^	<0.01
Radial Shoulder (R-SH)	166.96 (27.68) ^b A^		58.80 (12.53) ^a B^		161.62 (29.44) ^b A^	
Chamfer (CH)	167.96 (24.1) ^b A^		102.71 (18.01) ^b B^		163.3	
					(27.57) ^b A^	

SLM, Selective laser melting, LWT, lost wax technique, dissimilar superscript small alphabets in the same column denote significant difference (*p* < 0.05); dissimilar upper case superscript alphabets in the same row denote significant difference (*p* < 0.05) (Tukey–Kramer multiple comparisons test). * ANOVA.

**Table 3:** Mean and standard deviations of horizontal misfit (μm) among the study groups.

Margin Type	SLM	*p*-Value *	CAD-CAM	*p*-Value *	LWT	*p*-Value *
	Mean (SD) (μm)		Mean (SD) (μm)		Mean (SD) (μm)	
Shoulder (SH)	137.94	<0.05	135.00 (13.56) ^a A^	<0.05	100.00 (13.19) ^a a B^	0.09
	(37.85) ^a A^				93.93 (17.13) ^a B^	
Radial Shoulder (R-SH)	133.39 (20.87) ^ab A^		114.91 (15.29) ^ab AB^		89.38	
Chamfer (CH)	116.71 (27.67) ^b A^		95.31 (18.31) ^b AB^		(14.81) ^a B^	

SLM, Selective laser melting, LWT, lost wax technique, dissimilar superscript small alphabets in the same column denote significant difference (*p* < 0.05), dissimilar upper case superscript alphabets in the same row denote significant difference (*p* < 0.05) (Tukey–Kramer multiple comparisons test). * ANOVA.

## Data Availability

Data of the study are available through contact with the corresponding author.
